# An Obstacle-Tolerant Path Planning Algorithm for Mobile-Anchor-Node-Assisted Localization

**DOI:** 10.3390/s18030889

**Published:** 2018-03-16

**Authors:** Rong-Guei Tsai, Pei-Hsuan Tsai

**Affiliations:** Institute of Manufacturing Information and Systems, National Cheng Kung University, Tainan City 70101, Taiwan; P98031056@mail.ncku.edu.tw

**Keywords:** localization, mobile anchor node, path planning, wireless sensor networks

## Abstract

The location information obtained using a sensor is a critical requirement in wireless sensor networks. Numerous localization schemes have been proposed, among which mobile-anchor-node-assisted localization (MANAL) can reduce costs and overcome environmental constraints. A mobile anchor node (MAN) provides its own location information to assist the localization of sensor nodes. Numerous path planning schemes have been proposed for MANAL, but most scenarios assume the absence of obstacles in the environment. However, in a realistic environment, sensor nodes cannot be located because the obstacles block the path traversed by the MAN, thereby rendering the sensor incapable of receiving sufficient three location information from the MAN. This study proposes the obstacle-tolerant path planning (OTPP) approach to solve the sensor location problem owing to obstacle blockage. OTPP can approximate the optimum beacon point number and path planning, thereby ensuring that all the unknown nodes can receive the three location information from the MAN and reduce the number of MAN broadcast packet times. Experimental results demonstrate that OTPP performs better than Z-curves because it reduces the total number of beacon points utilized and is thus more suitable in an obstacle-present environment. Compared to the Z-curve, OTPP can reduce localization error and improve localization coverage.

## 1. Introduction

Wireless sensor networks (WSNs) consist of a number of sensing devices with external physical information and network communication capabilities. WSNs are deployed in an area to be detected, and they collect and report environmental information for users. The scope of application of WSNs includes the military, intelligent h, health care, and ecological detection [1]. In the application of WSNs, localization is a critical technique that enables unknown nodes (or ordinary nodes) to obtain their own location information to further optimize applications such as routing protocols and object tracking [2,3].

**Figure 1:**
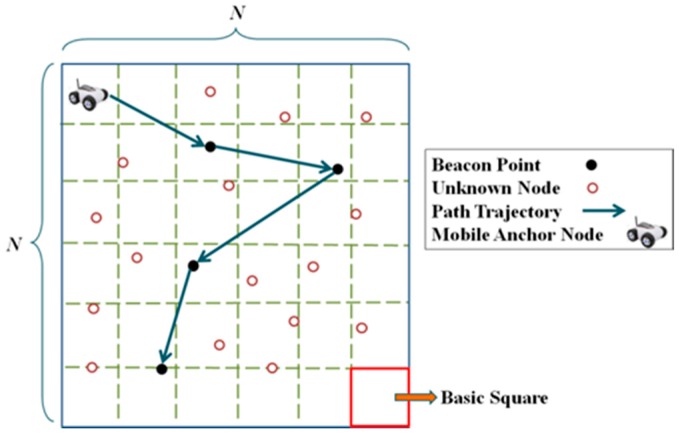
Network architecture of MANAL.

The Global Positioning System (GPS) is a tool that obtains location information. However, each unknown node equipped with a GPS transceiver will result in a substantial increase in costs. A viable method is utilizing a few anchor nodes. Anchor nodes are nodes that know their locations, being equipped with GPS transceivers. Anchor nodes broadcast messages including their location information to unknown nodes. Unknown nodes then use distance measurement technology to obtain the relative distance or angle relationship with the anchor nodes. Thereafter, the positions of these unknown nodes are calculated via localization algorithms [2,3,4,5,6]. The minimum number of required anchor nodes must ensure that each unknown node receives at least three messages from different anchor nodes, which is still costly. An alternative is using a dynamic anchor node, also known as a mobile anchor node (MAN), with a pre-planned path to continuously broadcast location information to replace multiple static anchor nodes. The locations where the MAN broadcasts messages are called beacon points. This localization architecture is called mobile-anchor-node-assisted localization (MANAL) [2]. MANAL comprises a GPS-equipped MAN and numerous unknown nodes [7,8], as shown in Figure 1.

Path planning is a critical issue in MANAL. The fundamental requirement for a MAN is to ensure that all the unknown nodes can receive three (non-colinear) packets, thereby enabling unknown nodes to calculate their own positions. Most environments have obstacles that involve communication-signal shadowing. For example, electromagnetic waves collide with obstacles on the transmission path, thereby resulting in reflection, diffraction, and scattering as well as in the unknown nodes being rendered incapable of receiving packets from a MAN.

To solve the problem of obstacle blocking, reference [8] proposed an intuitive method, in which a MAN changes its movement direction when it encounters obstacles, detours around the obstacles, and broadcasts packets to unknown nodes. However, this method cannot guarantee that all the unknown nodes will receive three packets for locating their position. In addition, a MAN is required to additionally broadcast substantial location information when changing the direction of movement, thereby resulting in excessive wastage of power for the MAN and unknown nodes.

To reduce costs and guarantee that all the unknown nodes in the sensing area receive at least three messages from MAN, there are optimal placements for beacon points. The optimal path of MAN is based on the optimal placement of beacon points. Obviously, the MAN’s path problem is one of the Hamiltonian path problems [9] that visits each vertex of the graph exactly once. The Hamiltonian path problem is a well-known NP-complete problem [10,11] and therefore MAN’s path is also an NP-complete problem. Therefore, this study presents an obstacle-tolerant path planning (OTPP) approach, which uses the divide and conquer strategy to divide the entire area into multiple unit squares, thereby optimizing the position of the beacon point and the required number of beacon points for each unit square. Lastly, all the beacon points are visited by MAN. To summarize, the contributions of this study are as follows:

1. In the obstacle-present environment, OTPP guarantees that all the unknown nodes can receive three location information (packets) from a MAN and the number of beacon points of MAN in OTTP is less than in Z-curve.
2. OTPP uses a rectangle as the boundary of each of the obstacles; any convex polygon is transformed into a rectangle, and two path trajectories for MAN are proposed.
3. In the obstacle-present environment, this study is the first to propose a static path planning method to optimize the number of beacon points. OTPP optimizes the number of beacon points, thereby reducing the number of broadcast times of MAN and receiving packet times of unknown nodes to facilitate the reduction in power consumption.
4. OTPP performs better than Z-curve because it uses less total number of beacon points and is thus more suitable in an obstacle-present environment. Compared to the Z-curve, OTPP can reduce localization error and improve localization coverage.
5. Online path planning methods cannot guarantee that all the unknown sensors in the sensing area can be positioned. Compared to them, our approach, OTPP, does not only guarantee full coverage of sensor localization but also has scalability and flexibility to adapt to environmental changes by adopting the divide-and-conquer method.

The rest of this paper is organized as follows: Section 2 reviews the related works and summarizes the existing path planning method for MANAL. Section 3 explains the problems for existing path planning in the obstacle-present environment. Section 4 presents the OTPP approach. Section 5 provides an analysis of the performance of the Z-curve, and OTPP through simulations, and Section 6 contains our conclusions and future works.

## 2. Related Works

Path planning algorithms can be categorized into two, namely *offline* [7,8,12,13,14,15,16,17,18] and *online* schemes [19,20,21,22,23,24,25,26,27]. The offline path planning schemes are based on conditional restrictions to determine the globally optimal path because a MAN visits the path and the location of the broadcast has been pre-planned. Thus, the computational capability of a MAN is low, and the number of broadcast times of a MAN can be optimized, thereby facilitating the reduction of energy consumption of the MAN and unknown nodes. The online path planning schemes are based on observable environments or sensor deployment situations. A MAN dynamically adjusts the feasible path to satisfy the system requirements, although online path planning has considerable flexibility. A MAN must have high computing capability because it must make decisions (e.g., move path trajectory or broadcast timing) during movements. In addition, the online path planning method can only provide the optimum solution locally, thereby failing to guarantee that all the unknown nodes can receive three location information of the MAN to estimate their locations [2]. Table 1 summarizes the comparison between the offline and online path planning algorithms for MANAL.

### 2.1. Offline Path Planning Algorithm

At present, most offline path planning algorithms assume an obstacle-free environment [7,12,13,14,15,16,17,18,19]. In [14], MAN comprises three anchor nodes and is based on the SCAN path trajectory to visit the entire sensing field. However, the construction cost of a MAN is substantially high. Han et al. proposed a localization algorithm with a mobile anchor node based on trilateration (LMAT), where the MAN moves in the trajectory of an isosceles triangle and sets the three corners of this triangle as the beacon points; thereby, solving the beacon point collinearity problem [15]. In [7], the three path planning schemes, namely SCAN, DOUBLE-SCAN, and Hilbert are compared. In the SCAN scheme, a MAN moves in a straight line along the *X*-axis (or *Y*-axis). Thus, the SCAN method covers the entire sensing field, and is convenient to implement. However, the beacon point collinearity problem remains unresolved in this scheme. In the DOUBLE-SCAN scheme, a MAN travels in straight lines along the *x*- and *y*-axes. Compared to SCAN, DOUBLE-SCAN has excessively long path lengths, thereby resulting in high energy consumption in the MAN and unknown nodes. Hence, the Hilbert method divides a sensing field into 4*^n^* sub-squares, and the central point of each sub-square is linked by 4*^n^* line segments, thereby generating non-collinear beacon points for MANs. Compared to the SCAN and DOUBLE-SCAN schemes, the Hilbert scheme substantially reduces the total path length of a MAN.

The CIRCLES and S-CURVES path-planning schemes were proposed in [12] to address the collinearity problem. In the CIRCLES scheme, the center of the sensing field is treated as the center of a circle, and a trajectory of a MAN is constructed thereafter from a few concentric circles of varying dimensions. The S-CURVES method is based on SCAN, although it alters the linear paths of a MAN into “S” shaped curves. Compared with SCAN, DOUBLE-SCAN, and Hilbert, the CIRCLES and S-CURVES methods can provide higher localization accuracy to the unknown nodes. Hu et al. proposed a scheme called mobile anchor centroid localization (MACL) [13], which is similar to CIRCLES because the former also uses the center of the sensing field as a center. However, MANs are assigned a spiral trajectory instead, thereby resulting in shorter total path lengths than those of the CIRCLES method.

The aforementioned offline path planning schemes assume an obstacle-free environment; however, only a few studies discuss the obstacle-present environment. Although Z-curve [8], perpendicular intersection (PI) scheme [16], and SCAN-based scheme [17] have been proposed as solutions for the obstacle-present problem, these schemes cannot guarantee that the unknown nodes in the area can receive three location-information from the MAN for localization. Guo et al. proposed a path planning scheme called PI [16], where a MAN is installed on a rotating arm and continuously broadcasts location information to unknown nodes based on RSSI and the MAN rotation angle. PI uses perpendicular intersection geometric relationship to calculate the location of the unknown nodes. In this method, the MAN requires continuous broadcast location information, thereby resulting in the high energy consumption of the MAN and unknown nodes. In [17], a range-free localization scheme is used, whereas a new path planning scheme based on SCAN is proposed in [18]. In view of the obstacle blocking problem, Ou et al. [17] propose the concept of using virtual keys, which can predict the location of the beacon point. SCAN-based schemes and PI exhibit identical drawbacks, and the MAN requires continuous broadcast location information. Rezazadeh et al. proposed a path planning method called Z-curve [8] to construct a “Z”-shaped path trajectory. The Z-curve can solve the beacon point collinearity problem, and is more effective than SCAN, Hilbert, and CIRCLES in terms of localization accuracy and time costs.

### 2.2. Online Path Planning Algorithm

The benefits of the online path planning algorithms are their environmental adaptability and flexibility because they do not require any prior knowledge of the environment. In addition, in the online path planning algorithms, the MAN’s path trajectory depends on the density of requests sent by unknown sensor nodes, and the MAN does not visit the entire area; therefore, online path planning algorithms are advantageous in terms of rapid positioning and power savings.

However, they are not suitable for applications that need full localization coverage. Online path planning algorithms are conducive to specific environments where sensors are distributed in special shapes (e.g., “C”- or “L”-shaped distributions) [19,20,21] and not isolated by obstacles [21,22,23]. However, for most applications in which unknown sensors are evenly distributed, online path planning algorithms fail to guarantee full coverage of sensors’ localization because the MAN’s path trajectory is not deterministic and depends on the density of requests sent by unknown sensor nodes [21,24,25]. In addition, the location of beacon points is not globally optimal, and therefore causes the unknown nodes to receive insufficient and inappropriate location messages, resulting in collinearity problems [26]. Therefore, online and offline path planning algorithms are applied to different scenarios.

Moreover, the online path planning algorithms are costly on hardware and communication. Hardware-wise, to implement online path planning algorithms, the MAN requires additional equipment. In [20], the MAN must configure directional antennas to receive and transmit signals in different directions and communicate with the unknown nodes within different directions in a region. In [27], the MAN was fixed with a pair of ultrasound transmitters. Each unknown node was assumed to be equipped with an ultrasound receiver at the two ends of a vehicle. Thus, the MAN behaved as a virtual ruler that traveled in the monitoring area to provide distance measurements to pairwise unknown nodes. The disadvantage of MAN in this case, is that it must configure a pair of ultrasound transmitters. In [19], a new method of mobile anchor group consisting of five mobile beacons, called maximum multi-hop of nodes (MMNs) was proposed. However, the MAN group compared to a single MAN is more expensive owing to the communication cost (CC) because unknown nodes proactively send a localization “request” message to the MAN in the process of positioning. Therefore, the unknown nodes consume a higher CC than the offline path planning algorithms [19,20,21].

Finally, a MAN must have high computing ability because it must make decisions (e.g., move paths or broadcast timing) during movements to deal with various situations, such as [21], where nodes would be clustered first, and MAN would visit these cluster heads based on the genetic algorithm. However, the computing complexity of MBL increases with the number of unknown nodes.

In this paper, we propose an offline path planning algorithm, which ensures that all the unknown nodes can receive three location-information from the MAN, and which optimizes the number of beacon points; therefore the MAN broadcast packet times and unknown node received packet times are reduced.

## 3. Overview

### 3.1. Preliminary

Table 2 presents the terminologies used in this paper.

**Figure 2:**
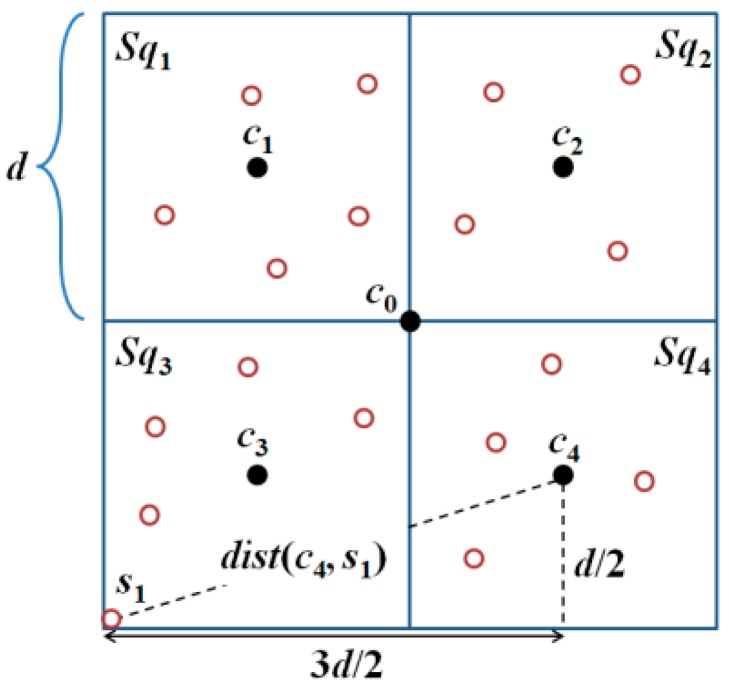
Communication radius, *CR*, must be larger than or equal to $\sqrt{\left(5/2\right)}d$ to ensure that all the unknown nodes in a basic square receive at least three position packets from the MAN.

**Definition** **1.** **Basic square.** *According to References* [7,8], *any N × N region can be partitioned into k basic squares. Each basic square must be divided into four sub-squares, Sq_h_, with a side length of d; where h ∈ {1, 2, 3, 4}, as shown in Figure 2.*

The locations of the beacon points of a MAN in a basic square are denoted by *c_0_*, *c*_1_, *c*_2_, *c*_3_, and *c*_4_, in Figure 2. Lemma 1 ensures that all the unknown nodes in a basic square can receive at least three position packets from the MAN.

**Lemma** **1.** An unknown node, s_i_, (i ∈ {1,2, …, n}), in a basic square can receive at least three position packets from the MAN if and only if $CR\geq \sqrt{\left(5/2\right)}d$, where CR represents the communication range of the MAN [7,8].

**Proof.**  This lemma originates from reference [7] where the detailed proof can be found. Here, we give a brief proof. Based on the localization method, an unknown node, *s*_1_, inside *Sq*_3_ can be localized only if it can receive three non-collinear position packets from the MAN. As $CR\geq \sqrt{\left(5/2\right)}d$, *dist*(*c*_0_, *s*_1_) ≤ $\sqrt{\left(5/2\right)}d$, *dist*(*c*_3_, *s*_1_) ≤ $\sqrt{\left(5/2\right)}d$, and *dist*(*c*_4_, *s*_1_) ≤ $\sqrt{\left(5/2\right)}d$, *s*_1_ can receive the position packets broadcast by the MAN at locations *c*_1_, *c*_3_, and *c*_4_, thereby being localized, as illustrated in Figure 2. Similarly, all the unknown nodes *s_i_* (*i* ∈ {1, 2, .., *n*}) that are inside *Sq*_1_, *Sq*_2_, and *Sq*_4_ can be localized.

The farthest location from the center of the basic square is the vertex of the square. Assume the unknown node *s*_1_ located at the vertex of the square can receive at least three position packets from the three nearest beacon points *c*_1_, *c*_3_, and *c*_4_, the communication range *CR* must be larger than Max[*dist*(*c*_0_, *s*_1_)], *dist*(*c*_3_, *s*_1_), *dist*(*c*_4_, *s*_1_)] and Max[*dist*(*c*_1_, *s*_1_)], *dist*(*c*_3_, *s*_1_), *dist*(*c*_4_, *s*_1_)] = $\sqrt{\left(5/2\right)}d$ so $CR\geq \sqrt{\left(5/2\right)}d$. ☐

### 3.2. Problem Description

**Figure 3:**
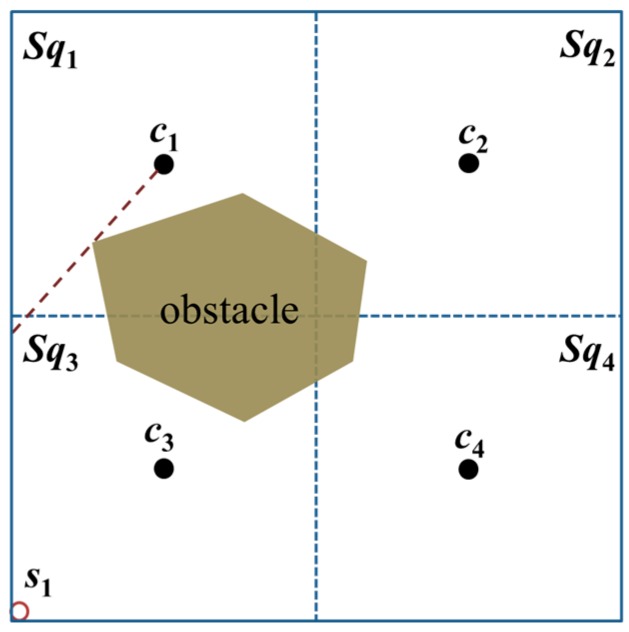
The unknown node *s*_1_ is blocked by an obstacle and thus cannot receive a minimum of three position packets from the MAN.

In an obstacle-present environment, assume that an obstacle exists between *s*_1_ and *c*_1_ as well as *s*_1_ and *c*_2_; then, *s*_1_ will receive only the position packets broadcast by the MAN at beacon points *c*_3_ and *c*_4_, as shown in Figure 3.

**Figure 4:**
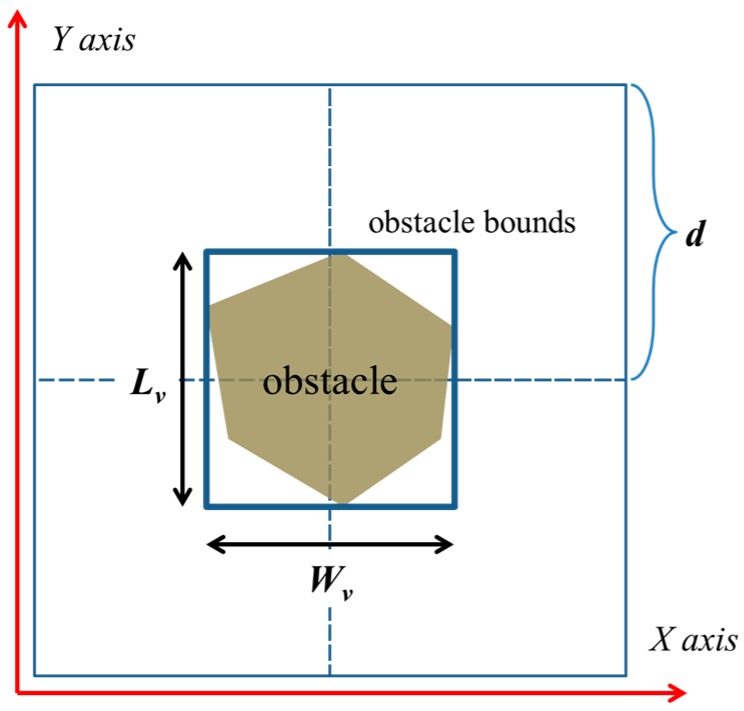
For simplicity, rectangles are used to bind obstacles in an obstacle-filled scenario.

Without loss of generality, in this work, an arbitrary convex polygonal obstacle is converted into a rectangle, as shown in Figure 4. *L_v_* is the edge of the obstacle and is parallel to the *Y*-axis; *W_v_* is the edge of the obstacle and is parallel to the *X*-axis. According to the relationship among *L_v_*, *W_v_,* and *d*, obstacles may be classified into the following three types:

$$\{\begin{array}{l} \mathrm{Type}\;I:\;L_{v}<d\;\mathrm{and}\;W_{v}<d.\\ \mathrm{Type}\;II:\;L_{v}<d\;\leq \;W_{v}.\\ \mathrm{Type}\;III:\;W_{v}<d\leq \;L_{v}. \end{array}$$

*k*-covered areas are defined to demonstrate the effects of an obstacle on unknown nodes in different areas.

**Definition** **2.** ***k*-covered area.** *An area is k-covered if all the unknown nodes within this area can receive k non-collinear position packets from the MAN.*

**Figure 5:**
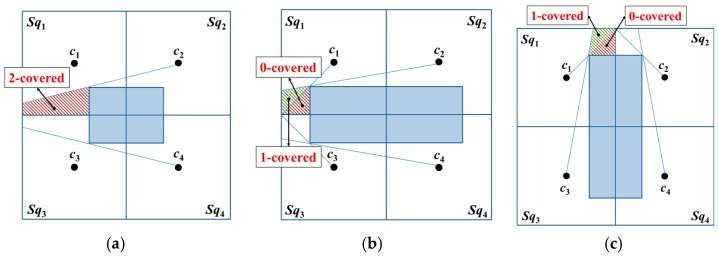
Obstacle shadowing results in the presence of non-three-covered areas in a basic square: (**a**) two-covered area, (**b**) one-covered and zero-covered areas where $L_{v}<d$ and *W_v_* ≥ d, and (**c**) one-covered and zero-covered areas where $\;L_{v}\geq d\;\mathrm{and}\;W_{v}<d$.

The following two scenarios are feasible in a basic square, in which the unknown nodes that are present in areas where *k* < 3 cannot be localized:

1. A two-covered area exists inside a basic square; the unknown nodes within this area can receive only two position packets from the MAN, as illustrated in Figure 5a.
2. One-covered and zero-covered areas are present within a basic square; the unknown nodes within these areas can receive only one and zero packets from the MAN, as shown in Figure 5b,c.

Evidently, additional beacon points are required to ensure that the entire basic square is a three-covered area.

## 4. Algorithm of OTPP

### 4.1. Deployment of Beacon Points in OTPP

Table 3 presents the flow of how to deploy the beacon points by OTPP which is coherent with the following lemmas. The following lemmas prove that OTPP guarantees full coverage of localization of unknown nodes.

**Definition** **3.** **Basic square with obstacles.** *Assume that each basic square contains a maximum of one obstacle with sides of lengths L_y_ and W_y_, and the center of the obstacle lies on the position of the beacon point c_0_.*

To determine the optimal placement of beacon points in a basic square with obstacles, we adopt divide-and-conquer approach. We first divide a basic square to four sub-squares because it is the maximum number of sub-squares which are identical and with minimum area size. To minimize the number of beacon points, the optimal location of beacon point should be able to cover all the unknown nodes in a sub-square. The union of optimal locations are called *reg*. As long as there are three non-collinear locations in *reg* selected as beacon points, all the unknown nodes can be localized in a sub-square. Accordingly, we can obtain the following Lemma 2.

**Lemma** **2.** If all the unknown nodes s_i_ in the sub-square can be localized, then, three non-collinear beacon points need to be present, and all the three beacon points will lie within reg_1_.

**Figure 6:**
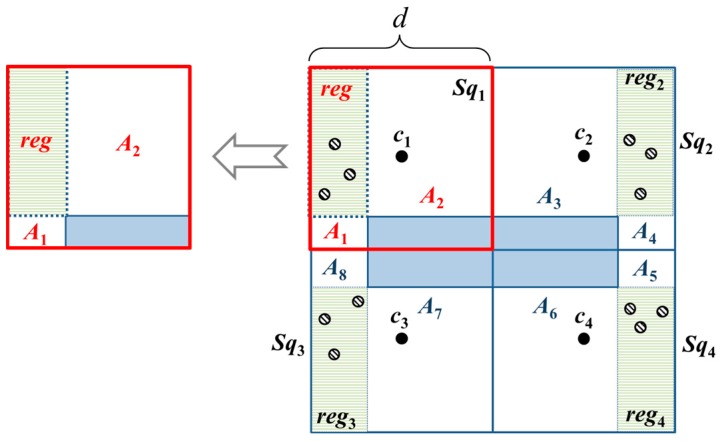
The layout of beacon points in the OTPP scheme and the dots are the possible locations of beacon points.

**Proof.**  The localization of an unknown node’s position requires at least three non-collinear beacon points [2]. Lemma 1 indicates that all the unknown nodes within the basic square can be localized. Therefore, the communication range (*CR*) of the MAN and unknown nodes are at least $\sqrt{\left(5/2\right)}d$. Since the four sub-squares are identical, we consider sub-square *Sq*_1_ as representative. In sub-square *Sq*_1_, the maximum distance is $\sqrt{2}d<\sqrt{\left(5/2\right)}d$. Hence, three beacon points are required to localize all the unknown nodes when no obstacles are present. When obstacles are present, to find *reg*, we extend the sides of the obstacle and hence the sub-squares are divided into three regions. *reg* is then bounded by the two lines from the extended sides of the obstacle. The other two regions, *A*_1_ and *A*_2_, cannot guarantee to cover all the unknown nodes in *Sq*_1_. To cover all unknown nodes in *A*_1_, the locations of three beacon points are bounded to the area on the left of the obstacle which are *A*_1_ and *reg*_1_. To cover all unknown nodes in *A*_2_, the locations of three beacon points are bounded to the area above the obstacle which are *A*_2_ and *reg*_1_. The intersection of the two areas is *reg*, as shown in Figure 6. ☐

We may obtain the following (Corollary 1) to enable the localization of all the unknown nodes *s_i_* within the entire basic square.

**Corollary** **1.** Given that each sub-square Sq_h_ (h ∈ {1, 2, 3, 4}) requires three beacon points, twelve beacon points should be added to cover the entirety of a basic square.

Beacon points should be added in *reg*_1_–*reg*_4_ owing to obstacle blocking and to ensure that each sub-square is a three-covered area. For a two-covered area, we can further obtain the following Lemma 3.

**Lemma** **3.** When a basic square contains two-covered areas, all the unknown nodes in a basic square can be localized, and a minimum of four beacon points should be added.

**Proof.**  When a basic square contains two-covered areas; the basic square can be divided into 12 regions based on the sides of the obstacle (i.e., *reg*_1_–*reg*_4_ and *A*_1_–*A*_8_), and two-covered areas lie within *A_i_*, *i* ∈ {1, 2, …, 8}. Therefore, adding a beacon point to reg_1_ results in the *A*_1_ and *A*_2_ regions receiving three beacon packets, thereby enabling the localization of the unknown nodes in these regions. Similarly, the addition of a beacon point to each of *reg*_2_–*reg*_4_ will enable each of the *A*_3_–*A*_8_ regions to receive three beacon packets and enable the localization of all the unknown nodes in these regions. Therefore, four beacon points should be added to enable the localization of all the unknown nodes within the entirety of the basic square. ☐

**Lemma** **4.** When a basic square contains zero-covered and one-covered areas, twelve beacon points should be added to the entirety of the basic square.

**Proof.**  The basic square contains zero-covered and one-covered regions, and can be divided into twelve regions based on the sides of the obstacle (i.e., *reg*_1_–*reg*_4_ and *A*_1_–*A*_8_), in which zero- and one-covered areas are present in *A*_1_, *A*_4_, *A*_5_, and *A*_8_. Meanwhile, two-covered regions are present in *A*_2_, *A*_3_, *A*_6_, and *A*_7_. Hence, the addition of three beacon points to reg_1_ will enable the *A*_1_ and *A*_2_ regions to receive three beacon packets and the localization of the unknown nodes within these regions. Similarly, the addition of three beacon points to each of *reg*_2_–*reg*_4_ enables each of *A*_3_–*A*_8_ to receive three beacon packets. Hence, the addition of twelve beacon points enables the localization of all the unknown nodes in the basic square. ☐

However, in Lemmas 3 and 4, which consider only the number of beacon points that should be deployed within each sub-square and do not consider the optimal deployment location, the excess number of beacon points will cause the MAN and unknown nodes to incur high energy cost.

**Lemma** **5.** When a basic square contains two-covered areas, a particular layout will enable two beacon points to completely cover the entirety of the basic square.

**Figure 7:**
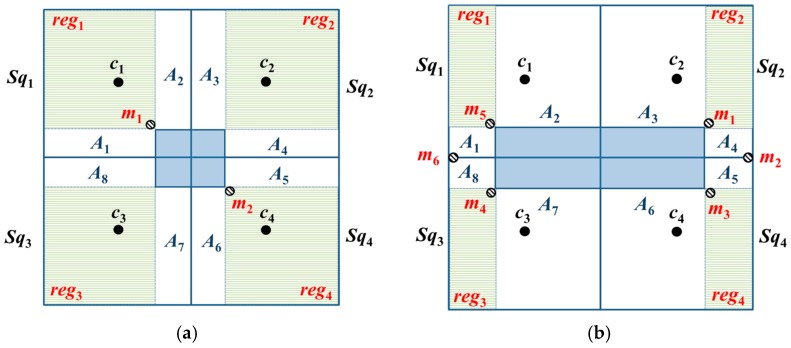
The results of beacon point simplification in scenarios where (**a**) the basic square contains two-covered areas and (**b**) the basic square contains zero-covered and one-covered areas.

**Proof.**  Lemma 3 indicates that the addition of a beacon point to each sub-square will enable complete coverage of the entirety of a basic square. However, this placement method does not consider the area that can be covered by a beacon point and only places a beacon point in each sub-square. Therefore, if we consider *CR* ≥ $\sqrt{\left(5/2\right)}d$, then, *A*_1_, *A*_2_, *A*_3_, and *A*_8_ can be covered straightforwardly by placing a beacon point at the position m_1_. In addition, all the unknown nodes in *A*_1_, *A*_2_, *A*_3_, and *A*_8_ will be able to receive at least three beacon packets. Similarly, by placing a beacon point at position m_2_, *A*_4_–*A*_7_ will be covered, and all the unknown nodes in *A*_4_–*A*_7_ will receive at least three beacon packets. Therefore, two beacon points should be added to completely cover the entirety of the basic square, as shown in Figure 7a. ☐

**Lemma** **6.** When a basic square contains zero- and one-covered areas, a particular layout will enable six beacon points to completely cover the entirety of the basic square.

**Proof.**  Lemma 4 indicates that the addition of three beacon points to each sub-square will cover the entirety of a basic square. However, this placement method does not consider the area that can be covered by the beacon point and only places three beacon points in each sub-square. Therefore, if we consider *CR* ≥ $\sqrt{\left(5/2\right)}d$, then, placing a beacon point in each of *m*_4_, *m*_5_, and *m*_6_ will cover *reg*_1_, *reg*_3_, *A*_1_, *A*_2_, *A*_7_, and *A*_8_. In addition, the unknown nodes in these regions will receive at least three beacon packets. Similarly, adding a beacon point to each of *m*_1_–*m*_3_ will cover *reg*_2_, *reg*_4_, and *A*_3_–*A*_6_; moreover, the unknown nodes in these regions will also receive at least three beacon packets. Therefore, the addition of six beacon points will enable complete coverage over the entirety of the basic square, as shown in Figure 7b. ☐

We can further calculate the total number of beacon points required for OTPP, *N_BP_*, as described by Lemma 7:

**Lemma** **7.** *In OTPP, the entire basic square contains only three-covered areas; the total number of beacon points, $N_{BP}$, required is five. The basic square contains two-covered areas; the total number of beacon points required is six; the basic square contains zero-covered and one-covered areas, and the total number of beacon points required is ten.*

(1) $$N_{BP}=\{\begin{array}{l} 5,\;\mathrm{when}\;\mathrm{three-covered}\\ 6,\;\mathrm{when}\;\mathrm{two-covered}\\ 10,\mathrm{when}\;\mathrm{one-coverd}\;\mathrm{and}\;\mathrm{zero-covered} \end{array}$$

**Proof.**  In an obstacle-free environment, the total number of beacon points, $N_{BP}$, required for OTPP is five (*c*_0_, *c*_1_, *c*_2_, *c*_3_, *c*_4_). When the entire basic square contains two-covered areas, which require adding two beacon points, the total maximum number of beacon points required is six (see Figure 7a). When the basic square contains zero-covered and one-covered areas, which require adding six beacon points, the total maximum number of beacon points required is ten (see Figure 7b). ☐

### 4.2. Path Planning in OTPP

The MAN must visit all the positions of the beacon points if the beacon points *c*_1_ and *c*_4_ were designated as its first (starting) point and final (ending) point positions for visitation. In the obstacle-free environment, the visit order of the beacon points for MAN is *c*_1_, *c*_2_, *c*_0_, *c*_3_, and *c*_4_.

1. Basic square containing two-covered areas

**Figure 8:**
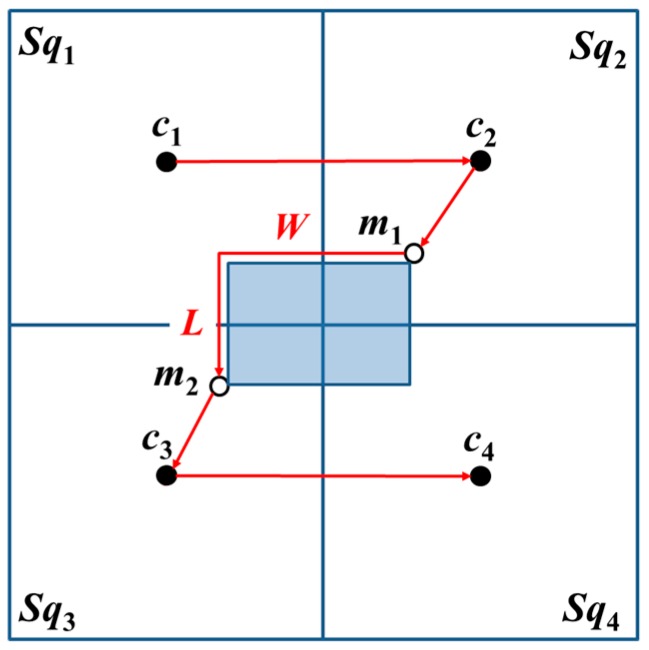
The trajectories of OTPP with basic square containing two-covered area. The red arrows are the trajectories while the shadow area is the obstacle.

When the basic square contains two-covered areas, the MAN first visits *c*_1_ and *c*_2_. From the four corners of an obstacle, the proximate location from *c*_2_ is selected, which is an additional beacon point *m*_1_, and its diagonal position is set as *m*_2_. Thereafter, the MAN visits *m*_1_ and *m*_2_. Lastly, the MAN visits *c*_3_ and *c*_4_, i.e., the MAN completes the travel on the entire basic square, as shown in Figure 8.

2. Basic square containing zero- and one-covered areas

**Figure 9:**
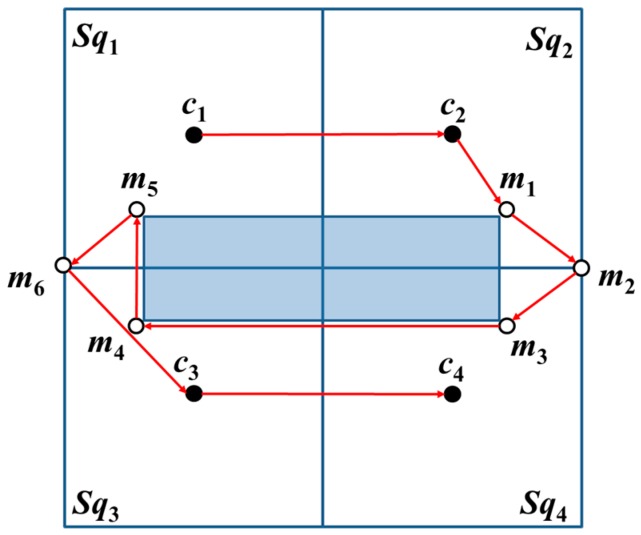
The straight-line path from *c*_1_ to *c*_2_ without blockage by an obstacle. The red arrows are the trajectories while the shadow area is the obstacle.

When the basic square contains zero-covered and one-covered areas, the MAN verifies whether the path from *c*_1_ to *c*_2_ is obstructed by an obstacle or if *c*_1_ to *c*_2_ has a straight path without being obstructed by an obstacle. The MAN first visits *c*_1_ and *c*_2_. Among the additional beacon points *m_q_* (*q* = 1, 2, …, 6), the additional beacon point proximate to *c*_2_ is selected as the next beacon point to be visited following the visit to *m*_1_–*m*_6_. Lastly, the MAN visits *c*_3_ and *c*_4_, as shown in Figure 9.

**Figure 10:**
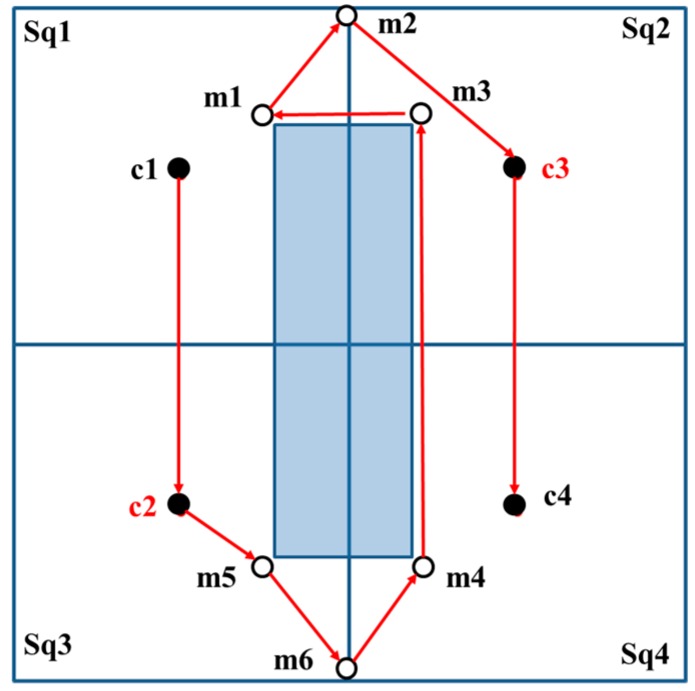
The straight-line path from *c*_1_ to *c*_2_ is blocked by an obstacle; therefore, swap the positions of *c*_2_ and *c*_3_. The red arrows are the trajectories while the shadow area is the obstacle.

In another case, if the straight-line path from *c*_1_ to *c*_2_ is blocked by an obstacle, then, the positions of *c*_2_ and *c*_3_ are interchanged. The MAN first visits *c*_1_ and *c*_2_. Thereafter, additional beacon points *m_q_*, proximate to *c*_2_ are selected as the beacon points to be visited next. The sequential visits to the additional beacon points follow the order *m*_5_, *m*_6_, *m*_4_, *m*_3_, *m*_1_, and *m*_2_. Lastly, the MAN visits *c*_3_ and *c*_4_, as shown in Figure 10. Figure 10 is considered as the result of the rotation of Figure 9 by 90°.

## 5. Performance Evaluation

The performance of OTPP is evaluated by three metrics (1) number of beacon points, (2) average localization errors, and (3) localization coverage. In Section 5.1, we describe the three metrics and compare three common localization techniques. The settings of experimental parameters are introduced in Section 5.2. Finally, we change the shapes of obstacles and the communication range to see the impact of blocking rate and the impact of resolution. The results are presented in Section 5.3.

### 5.1. Evaluation Metrics and Localization Techniques

1. *Number of beacon points*: The number of beacon points stand for the number of times that the MAN broadcasts packets. The more the times of MAN broadcasting, the more the packets the unknown nodes receive. An increase in the number of packets received by an unknown node, leads to a corresponding increase in power consumption per unknown node so that the performance of entire sensor networks, decreases.
2. *Average localization error*: Localization error is defined as the difference between the estimated locations and actual locations of unknown nodes. Equation (2) presents the localization error of an unknown node $s_{i}$, where $\left(x_{i},y_{i}\right)$ is the coordinate of the actual location and ($x_{esti},y_{esti})$ is the coordinate of the estimated location. Average localization error is presented in Equation (3) where *n* is the total number of unknown nodes. The lower the average localization errors, the better the performance of sensor networks:
  (2) $$error\left(s_{i}\right)=\sqrt{{\left(x_{i}-x_{esti}\right)}^{2}+{\left(y_{i}-y_{esti}\right)}^{2}}$$
  (3) $$AvgError=\frac{\sum_{i=1}^{n}error\left(s_{i}\right)}{n}$$
3. *Localization coverage*: An unknown node can successfully do self-positioning as long as it receives three or more packets from the MAN. The more the unknown nodes successfully self-positions, the better the performance of sensor networks. Therefore, we define the localization coverage as the ratio of the number of unknown nodes that receive three or more MAN packets to the total number of unknown nodes [8].

There are three common localization techniques, weighted centroid localization (WCL) [28], accuracy priority trilateration (APT) [8], and time priority trilateration (TPT) [8]. The descriptions are as follows:

(A) *Weighted Centroid Localization* (WCL): WCL defines a weight function $RSS_{ij}$ which is the RSS value of the MAN at different beacon points to indicate the impact of different beacon point coordinates *b_j_*(*x*, *y*). Equation (4) expresses how an unknown sensor, *s_i_* calculates its own positions, *P*(*s_i_*) based on averaging the coordinates of all *N_r_* received beacon points.
  (4) $$P\left(s_{i}\right)=\frac{\sum_{j=1}^{N_{r}}RSS_{ij}\times b_{j}}{\sum_{j=1}^{N_{r}}RSS_{ij}}$$
(B) *Time-Priority Trilateration* (TPT): TPT calculation starts as soon as three localization packets are received. This enables sensors to save power and the time they need to spend waiting. With these localization packets, the following system of equations needs to be solved to obtain the localization result. For computing the position of *s_i_*(*x_i_*, *y_i_*) in a 2-D space, the following system of equations must be solved:
  (5) $$P\left(s_{i}\right)=\{\begin{array}{c} dist\left(r_{1},s_{i}\right)=\sqrt{{\left(x_{1}-x_{i}\right)}^{2}+{\left(y_{1}-y_{i}\right)}^{2}}\\ dist\left(r_{2},s_{i}\right)=\sqrt{{\left(x_{2}-x_{i}\right)}^{2}+{\left(y_{2}-y_{i}\right)}^{2}}\\ dist\left(r_{3},s_{i}\right)=\sqrt{{\left(x_{3}-x_{i}\right)}^{2}+{\left(y_{3}-y_{i}\right)}^{2}} \end{array}$$
(C) *Accuracy-Priority Trilateration* (APT): Accuracy-Priority Trilateration (APT) is very similar to TPT. The difference between APT and TPT is that APT checks whether to start the trilateration calculation within a period of time. If a sensor has received at least three localization packets, it starts the trilateration calculation with three of the nearest keys.

**Figure 11:**
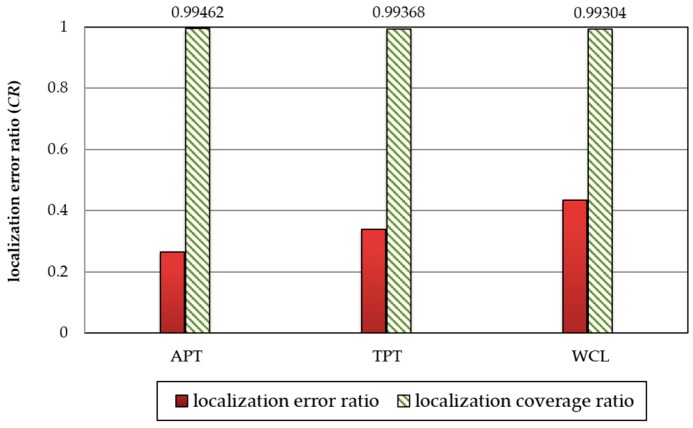
Comparison of APT, TPT, and WCL techniques in terms of localization error and coverage.

In Figure 11, the experimental results show that the localization errors of APT, TPT, and WCL are 0.265, 0.339, and 0.434 *CR*, respectively while their corresponding localization coverages are similar. Therefore, we adopt the APT technique in the following experiments because APT is more accurate than TPT and WCL. The experimental parameter settings are shown in Table 4.

### 5.2. Simulation Setup

The first experiment aims to investigate the impact of blocking rate which is the ratio of the obstacle area to the entire area. We increase the rate from 0% to 30%. The second experiment aims to investigate the impact of the resolution, which is *CR/d*. We assume that the entire area size is 128 m×128 m, and *d* is fixed at 16 m. The number of MAN is one. The total number of unknown nodes is 500, and the communication range *CR* of the first experiment is $\sqrt{5}\times d$. The wireless channel model is the log-normal model [8,16,29], as shown in Equations (6) and (7), where, $P_{rec}$ denotes the received power, while $P_{trans}$ and $P_{loss}$ denote the transmission power of the MAN and the power attenuation during transmission, respectively. $X_{\sigma }$ is the Gaussian random variable where the standard deviation and mean are set to 2 and 0 according to the environment, which has different set parameters [16]. All the experiments are implemented in C++ programming language and all the results are the average of 100 simulations. Table 4 shows the experimental parameters that reference the Z-curve [8], which is the compared target in this paper:

(6) $$P_{rec}=P_{trans}-P_{loss}$$

(7) $$P_{loss}\left(d\right)=P_{loss}\left(d_{0}\right)+10\gamma \log\left(\frac{d}{d_{0}}\right)+X_{\sigma }$$

### 5.3. Simulation Results

#### 5.3.1. Impact of Blocking Rate

In an obstacle-free environment, OTPP performs as the Z-curve. The MAN travels the entire basic square with five beacon points. However, in the obstacle-present environment, the number of beacon points varies with the shapes of obstacles. Without loss of generality, shapes of obstacles are categorized into three types which are described in Section 3.2. Table 5 displays the experimental results which show the average number of beacon points needed by OTPP and Z-curve for different types of obstacles. The average number of beacon points needed by OTPP is observed to be less than that needed by the Z-curve in all obstacle-present environments.

**Figure 12:**
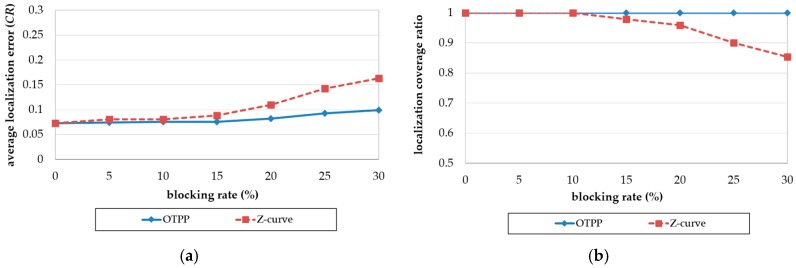
Impact of blocking rate on (**a**) localization error and (**b**) coverage ratio.

Figure 12a is the result of comparing the Z-curve and OTPP, with respect to the impact of blocking rate on localization error. Both the localization errors of OTPP and Z-curve increase with an increase in blocking rate. The higher the blocking rate, the higher the difference between the localization errors of the Z-curve and OTPP schemes. The difference is more obvious when the blocking rate is higher than 10%. When the blocking rate is 30%, the difference between the localization errors reaches a value of 0.072 *CR*.

Figure 12b is the result of comparing the Z-curve and OTPP, with respect to the impact of blocking rate on localization coverage. The coverage of the Z-curve decreases with an increase in the blocking rate while OTPP maintain almost full coverage. When the ratio is 30%, the difference in localization coverage between the Z-curve and OTPP reaches 15%.

**Figure 13:**
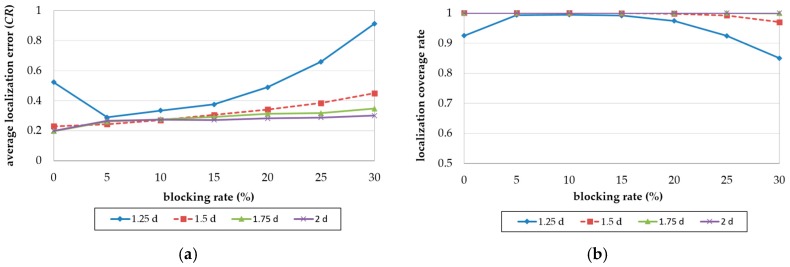
Impact of blocking rate on (**a**) localization error and (**b**) coverage ratio.

We then analyzed the OTPP in terms of the localization accuracy and coverage with a different communication range by fixing *d* as 16 m and changing the resolutions (*CR*/*d*). Figure 13a,b illustrate the localization accuracy and coverage with different resolution values (*CR*/*d* = 1.25*d*, 1.5*d*, 1.75*d*, and 2*d*, respectively), in OTPP.

In an obstacle-free environment which is blocking rate = 0, the localization accuracy and coverage ratio with resolution = 1.25*d* is lower than the others because its communication range is less than 1.6d which violates lemma 1, resulting in insufficient location messages received by unknown nodes. When the resolution is higher than 1.6 *d*, the localization error then decreases to 0.2 *CR* and the coverage ratio is 100%*.* As the blocking rate of obstacles increases, the beacon points are added in OTPP according to the obstacle types and therefore shorten the distance between unknown nodes and beacon points so the localization error with resolution 1.25*d* and blocking rate decreases and the coverage ratio increases when blocking rate is under 5%. As the blocking rate increases to more than 5%, the localization accuracy and coverage both increase. However, for those whose resolution > 1.6*d*, their localization error and coverage are almost not affected by blocking rates.

In summary, compared to Z-curve, OTPP has less number of beacon points, lower localization errors and higher localization coverage. OTPP therefore performs better in obstacle-present environments. When the communication range $CR\geq \sqrt{\left(5/2\right)}d$, 100% localization coverage ratio is achieved.

#### 5.3.2. Impact of Resolution

**Figure 14:**
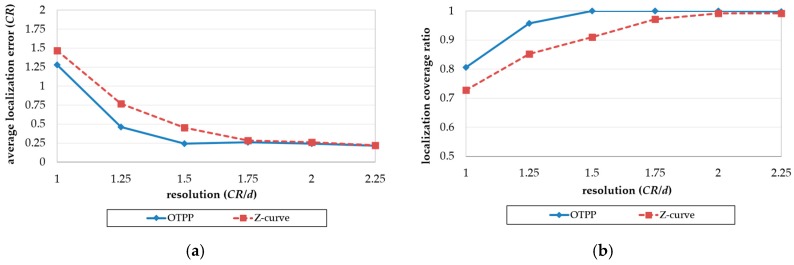
Impact of resolution on (**a**) localization error and (**b**) coverage ratio.

Figure 14a,b show the impact of resolution (*CR*/*d*) on localization errors and coverage. The field size is 128 m × 128 m and the communication range increases from *d* to 2.25*d*. According to Lemma 1, the minimum communication range, *CR* must be equal to or larger than $\sqrt{\left(5/2\right)}d\;≅1.6d$ to ensure that all unknown nodes in a basic square can receive at least three position packets from the MAN in an obstacle-free environment. In Figure 14a, with an increase in resolution, the localization errors of the OTPP decreases together with that of the Z-curve, with both stabilizing when the resolution is larger than 1.6*d*. OTPP performs better than Z-curve with respect to localization errors even though the resolutions are not large enough to fully cover a basic square. In Figure 14b, with an increase in resolution, the coverage of the OTPP increases together with that of the Z-curve. The coverage of the OTPP stabilizes when resolution is sufficient (≅1.6d) but the coverage of the Z-curve does not stabilize until the resolution is ≥2d.

#### 5.3.3. OTPP vs. Online Path Planning Algorithm

**Figure 15:**
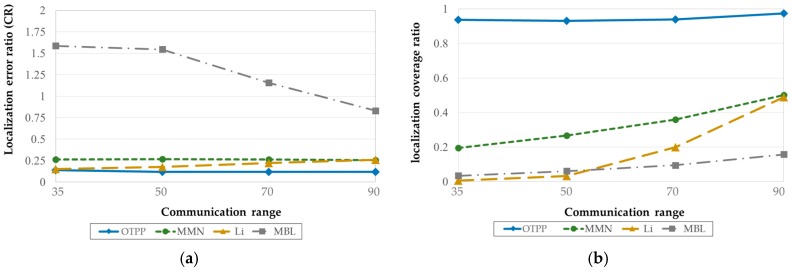
OTPP compared with online path planning in terms of (**a**) localization error and (**b**) coverage ratio. The unit of communication range is meter (m).

Figure 15a,b show the OTPP compared with online path planning algorithms in terms of localization errors and coverage. The online path planning algorithms of interest are MMN [19], Li [20], and MBL [21], with unknown nodes randomly deployed over a square sensing field of 1000 m×1000 m, and communication range, set to 35 m, 50 m, 70 m, and 90 m, respectively. It is observed that the localization error of the OTPP ≅ 0.2*CR* is less than that of MBL, MMN, and Li. Although the localization accuracy of MMN and Li are close to that of OTPP, the localization coverage of these online path planning algorithms is less than 50%, whereas OTPP can achieve ≅95%. As the communication range increases, the coverage area is also improved (e.g., MBL); however, the MAN will determine the direction and path of the trajectory based on the number of location request messages from unknown nodes, and there is no guarantee that all the unknown nodes can receive three packets from the MAN.

## 6. Summary and Future Work

Mobile-anchor-node-assisted localization (MANAL) has becomes more popular because it not only saves the power consumption of unknown nodes in sensor networks, but also adapts to various environments. The key problem of MANAL is the path planning of the MAN. Many path planning schemes have been proposed for MANAL; however, most of them only adapt to obstacle-free environments, which is not practical. In this paper, we propose an obstacle-tolerant path planning algorithm, called OTPP. Compared to the other path planning algorithms for MANAL, OTPP ensures that all unknown nodes are covered by at least three beacon points by using minimum beacon points in a unit square area. The experimental results show that OTPP performs better than the Z-curve as the total number of beacon points is reduced, localization errors lowered and coverage increased. This implies that OTPP is more adaptive to obstacle-present environments. In the future, we will consider more practical environments, such as obstacles with mobility or 3D spaces in path planning for application in unmanned aerial vehicles or detection of marine resources.

## Figures and Tables

**Table 1:** Comparison of path planning algorithms.

Path Planning Algorithm	Type	NM	Localization Method	CC	AC	Accuracy
SCAN [7], DOUBLE-SCAN [7], Hilbert [7]	Offline	Single	Range-based	Low	No	Low
Z-curve [8]	Offline	Single	Range-based	Low	Yes	High
CIRCLES and S-CURVES [12]	Offline	Single	Range-based	Low	No	Low
MACL [13]	Offline	Single	Range-based	Low	No	Low
GMAN [14]	Offline	Multiple	Range-based	Low	Yes	High
LMAT [15]	Offline	Single	Range-based	Low	Yes	High
PI [16]	Offline	Single	Range-free	Low	Yes	High
SCAN-based [17]	Offline	Single	Range-free	Low	Yes	High
MMN [19]	Online	Multiple	Range-based	High	No	Low
Li [20]	Online	Single	Range-based	High	No	Low
MBL (ndc) [19]	Online	Single	Range-based	High	No	Low
MBAL [22]	Online	Single	Range-based	High	No	Low
DREAMS [23]	Online	Single	Range-based	High	No	Low
Visibility Binary Tree (VBT) [24]	Online	Single	Range-based	High	No	Low
BRF and BTG [25]	Online	Single	Range-based	High	No	Low
Snake-like [26]	Online	Single	Range-based	High	No	Low
Virtual Ruler [27]	Online	Multiple	Range-based	High	No	Low

Note: NM—Number of MAN; CC—Communication Cost; AC—Area Full Coverage.

**Table 2:** Terminologies.

Terminologies	Definition and Description
Unknown (Ordinary) Node	The ordinary sensor nodes are used to finish the sensing data collection. The ordinary nodes are assumed to have less battery energy and keep silent state unless the broadcast beacon packet from MAN wakes them up, then estimate their coordinates.
Mobile Anchor Node (MAN)	The node with more energy, larger communication and mobility, is aware of its position by GPS or manually placed to give help to locate the ordinary nodes.
MANAL	Mobile-anchor-node-assisted localization.
Beacon Packet (packet)	A packet with position information broadcast by MAN.
Beacon Point	The location where the mobile anchor node broadcasts packets.
Basic Square	The minimum sensing field in OTPP.
Sub Square	The basic unit of basic square
*k*-covered area	An area can receive *k* non-collinear beacon packets from the MAN
OTPP	Obstacle-tolerant path planning

**Table 3:** Deployment of beacon points in OTPP.

OTPP Beacon Point Deployment Algorithm	
Input: Given an N×N sensing field and the communication range of MAN, *CR*.	
Output: Deployment of beacon points	
1:	Compute the maximum side length, *d*, of a basic square based on $\mathrm{CR}\geq \sqrt{5/2}d$.
2:	Compute the minimum number of basic squares to cover the sensing field.
3:	Segment the sensing area to multiple basic squares.
4:	For each basic square segmentation in the sensing area
i:	Analyze the composition (*k*-covered area) of the basic square.
ii:	Add beacon points into the basic square according to *k*-covered area.

**Table 4:** Experimental parameters.

Parameter	Value
Field Size	32 m×32 m, 128 m×128 m, 1000 m×1000 m
Number of Unknown Nodes (*N_S_*)	500
Number of MAN	1
Communication range (*CR*)	$\sqrt{5}\;$*d*, d,1.25d, 1.5d,1.75d, 2d,2.25d
Transmission Power (*P_trans_*)	250 dBM
Path Loss (*γ*)	3.3
Power Loss *P_L_*(*d*_0_)	55 dB
*d*_0_	1
Simulation Run	100

**Table 5:** Average number of beacon points.

Types of Obstacle	OTPP	Z-Curve
Obstacle-free	5	5
Type I	6	8
Type II	10	16
Type III	10	16

## Appendix A

**Table A1:** Values of Figure 12 (Average localization error/Standard deviation).

Blocking Rate (%)	OTPP	Z-Curve
0%	0.072642/0.002473	0.072548/0.002266
5%	0.07423/0.002835	0.080395/0.003239
10%	0.075144/0.003157	0.080409/0.003066
15%	0.075517/0.003261	0.088176/0.002725
20%	0.082042/0.003323	0.109765/0.002742
25%	0.092433/0.004709	0.142201/0.005814
30%	0.099022/0.004542	0.162859/0.007393

**Table A2:** Values of Figure 13 (Average localization error/Standard deviation).

Blocking Rate (%)	*CR* = 1.25*d*	*CR* = 1.5*d*	*CR* = 1.75*d*	*CR* = 2*d*
0%	0.522736/0.011586	0.229271/0.004582	0.196305/0.002656	0.198529/0.002546
5%	0.288306/0.006262	0.243143/0.004956	0.260096/0.003309	0.265602/0.002699
10%	0.333439/0.006373	0.269417/0.004646	0.275515/0.003111	0.272391/0.002974
15%	0.374983/0.007359	0.304929/0.005416	0.290966/0.004587	0.270626/0.003400
20%	0.488731/0.009895	0.341111/0.005022	0.311428/0.004127	0.281862/0.003496
25%	0.658398/0.011234	0.383832/0.006595	0.316821/0.005444	0.286928/0.004905
30%	0.913244/0.013579	0.449443/0.007821	0.346859/0.006346	0.300728/0.005595

**Table A3:** Values of Figure 14 (Average localization error/ Standard deviation).

Resolution (*CR*/*d*)	OTPP	Z-Curve
1*d*	1.279855/0.021720	1.465825/0.025145
1.25*d*	0.463241/0.011367	0.766297/0.018753
1.5*d*	0.245316/0.007551	0.455239/0.013257
1.75*d*	0.262951/0.006284	0.283528/0.011040
2.*d*	0.242452/0.005415	0.259893/0.010880
2.25*d*	0.215318/0.005089	0.219514/0.008451
